# Burden, risk factors, neurosurgical evacuation outcomes, and predictors of mortality among traumatic brain injury patients with expansive intracranial hematomas in Uganda: a mixed methods study design

**DOI:** 10.1186/s12893-023-02227-9

**Published:** 2023-10-25

**Authors:** Larrey Kasereka Kamabu, Godfrey S. Bbosa, Hervé Monka Lekuya, Eugene J. Cho, Victor Meza Kyaruzi, Arsene Daniel Nyalundja, Daniel Deng, Juliet Nalwanga Sekabunga, Louange Maha Kataka, Doomwin Oscar Deogratius Obiga, Joel Kiryabwire, Martin N. Kaddumukasa, Mark Kaddumukasa, Anthony T. Fuller, Moses Galukande

**Affiliations:** 1 Department of Surgery, Neurosurgery, College of Medicine, Makerere University, Kampala, Uganda; 2 Faculty of Medicine, Université Catholique du Graben, Butembo, Democratic Republic of the Congo; 3 Department of Surgery, Makerere University College of Health Medicine, Mulago Upper Hill, Kampala, Uganda; 4 Department of Pharmacology & Therapeutics, Makerere University College of Health Sciences, Kampala, Uganda; 5 Directorate of Surgical Services, Neurosurgical Unit, Mulago National Referral Hospital, Kampala, Uganda; 6 Department of Human Structure & Repair/ Neurosurgery, Faculty of Medicine, Ghent University, Ghent, Belgium; 7 Duke University, Durham, NC USA; 8 Department of Surgery, School of Medicine, Muhimbili University of Health and Allied Sciences, Dar es Salaam, Tanzania; 9 Faculty of Medicine, Université Catholique de Bukavu, Bukavu, South Kivu Democratic Republic of the Congo; 10 Duke Global Neurosurgery, Neurology and Health System, Duke University, Durham, NC USA; 11 Department of Medicine, School of Medicine, College of Health Sciences, Makerere University, P.O. Box 7072, Kampala, Uganda

**Keywords:** Traumatic expansive intracranial hematomas, Burden, Risk factors, Neurosurgical outcomes, And predictors of mortality

## Abstract

**Background:**

Expansive intracranial hematomas (EIH) following traumatic brain injury (TBI) continue to be a public health problem in Uganda. Data is limited regarding the neurosurgical outcomes of TBI patients. This study investigated the neurosurgical outcomes and associated risk factors of EIH among TBI patients at Mulago National Referral Hospital (MNRH).

**Methods:**

A total of 324 subjects were enrolled using a prospective cohort study. Socio-demographic, risk factors and complications were collected using a study questionnaire. Study participants were followed up for 180 days. Univariate, multivariable, Cox regression analyses, Kaplan Meir survival curves, and log rank tests were sequentially conducted. *P*-values of < 0.05 at 95% Confidence interval (CI) were considered to be statistically significant.

**Results:**

Of the 324 patients with intracranial hematomas, 80.6% were male. The mean age of the study participants was 37.5 ± 17.4 years. Prevalence of EIH was 59.3% (0.59 (95% CI: 0.54 to 0.65)). Participants who were aged 39 years and above; PR = 1.54 (95% CI: 1.20 to 1.97; *P* = 0.001), and those who smoke PR = 1.21 (95% CI: 1.00 to 1.47; *P* = 0.048), and presence of swirl sign PR = 2.26 (95% CI: 1.29 to 3.95; *P* = 0.004) were found to be at higher risk for EIH. Kaplan Meier survival curve indicated that mortality at the 16-month follow-up was 53.4% (95% CI: 28.1 to 85.0). Multivariate Cox regression indicated that the predictors of mortality were old age, MAP above 95 mmHg, low GCS, complications such as infection, spasticity, wound dehiscence, CSF leaks, having GOS < 3, QoLIBRI < 50, SDH, contusion, and EIH.

**Conclusion:**

EIH is common in Uganda following RTA with an occurrence of 59.3% and a 16-month higher mortality rate. An increased age above 39 years, smoking, having severe systemic disease, and the presence of swirl sign are independent risk factors. Old age, MAP above 95 mmHg, low GCS, complications such as infection, spasticity, wound dehiscence, CSF leaks, having a GOS < 3, QoLIBRI < 50, ASDH, and contusion are predictors of mortality. These findings imply that all patients with intracranial hematomas (IH) need to be monitored closely and a repeat CT scan to be done within a specific period following their initial CT scan. We recommend the development of a protocol for specific surgical and medical interventions that can be implemented for patients at moderate and severe risk for EIH.

**Supplementary Information:**

The online version contains supplementary material available at 10.1186/s12893-023-02227-9.

## Introduction

Expansive intracranial hematomas (EIH) due to traumatic brain injury (TBI) following road traffic accidents (RTA), assaults, and falls are a major public health problem worldwide [1]. EIH refers to evidence of an increased hematoma volume of more than 33% within the intracranial vault or absolute hematoma growth of more than 6 mL based on the initial CT scan with varying complications [2]. These injuries frequently worsen because of ongoing pathophysiological processes in the brain of the TBI patients leading to the development of EIH. EIH is a serious medical condition characterized by bleeding within the skull, leading to increased pressure on the brain. When not promptly addressed, EIH can result in life-threatening complications. Failure to intervene promptly can lead to neurological deterioration and, in extreme cases, death. Rapidly assessing and diagnosing EIH is critical to avoid its potentially devastating consequences. Previous reports and observations have reported several factors contributing to EIH following TBI, including age, mechanism of trauma, hypoxia, prehospital systolic blood pressure, initial hematoma volume, and location [3–6]. When prompt interventions are not put into place, EIH is harmful to the central nervous system by causing edema, ischemia, increased intracranial pressure, and brain herniations. EIH that follows TBI is caused by several reasons, although the risk factors for these patients in Uganda are not well understood [7]. EIH is the deadliest cerebral hematoma among the different intracranial bleeding subtypes that develop within the first 24 h following TBI [8–11] and to some patients, it worsens after 3–4 days [9, 12]. Globally, 16 to 75% of TBI patients experience EIH on subsequent CT scans with varying consequences [13]. However, there is no EIH therapy that has been reported to be more effective large-scale clinical trials [14–18]. Previous reports and observations [19] have revealed an increased prevalence of intracranial hematomas among TBI patients admitted to the Haukeland University Hospital in the country-wide hospitals in Norway following RTA, assaults, and falls.

Mulago National Referral Hospital (MNRH), a prominent facility treating traumatic brain injuries, has witnessed the significant impact of EIH on TBI patients. At the MNRH, TBI is the second most reported form of trauma, accounting for 27%, just after orthopedics at 37% [20]. According to the monthly reports from the casualty registry, casualty theatre, and main operating theatre, TBI admissions and deaths rank higher than orthopedics. In accident and emergency unit, TBI accounts for 75% of the fatalities [21]. It has been reported that MNRH admitted a total of 3,866 patients in casualty during the first quarter of 2018, and of these over 827 patients (21%) had TBI; 130 (15.7%) had a clinical radiological diagnosis of acute subdural or extradural hematoma thus increasing the number of craniotomies and craniectomies for extra-axial hematoma by threefold in 1 year [21]. Monthly, the Accident and Emergency ward attends to 64 urgent surgical evacuation of expansive hematomas in patients with acute TBI. Due to the high prevalence of traumatic brain injuries, the hospital frequently encounters cases of EIH. Previous studies have shown that TBI patients with some presenting with EIH report to the MNRH emergency unit at different time periods following an accident, thus experiencing varying surgical evacuation outcomes [17–19]. The dire consequences of EIH on TBI patients at MNRH highlight the urgent need for prompt interventions.

Furthermore, the lack of evidence-based protocols for the management and monitoring of neurosurgical outcomes, as well as delays in administration and the administration of appropriate interventions have led to unfavorable outcomes for some patients. In addition, TBI patients normally arrive at the MNRH emergency unit and other hospitals within 24 h, though occasionally some arrive later (3 to 4 days) with already EIH developed. However, there is insufficient information on the burden of EIH among TBI patients with IH presenting at the emergency unit at MNRH, the risk factors leading to the development of EIH among these patients, and the surgical outcomes. Many TBI patients with EIH, often die before interventions are instituted or during intervention due to inadequate healthcare facilities, limited theatre access, inadequate postoperative care, few neurosurgeons and anesthetists, delayed decision making, and lack of scientific up-to-date information and evidence on management of such patients in Uganda and many other developing nations worldwide [1, 22, 23]. Given the high rate and early time course of this deleterious form of intracranial hematoma, identifying patient demographics, as well as clinical, laboratory, and imaging characteristics is crucial to inform community-based interventions [13]. EIH leads to clinical deterioration and death if not managed urgently through timely surgical evacuation which is critical [13, 24].

Despite the critical nature of EIH, MNRH faces several challenges in ensuring timely interventions. Lack of resources, funding, limited staff, high patient expenses, fluctuating motivation, the financial barrier of purchasing surgical supplies, and access to medicine and advanced imaging equipment are all factors that can lead to delays in diagnosis and treatment thus further affecting patient outcomes. The need for heightened awareness and improved healthcare infrastructure is evident to optimize patient outcomes. The study investigated the burden, risk factors, surgical evacuation outcome, and predictors of mortality of EIH following TBI at the MNRH.

## Material and methods

### Study design

A mixed methods study design was used to investigate the burden, risk factors, surgical evacuation outcome, and predictors of mortality of EIH following TBI between the 16^th^ of June 2021 and the 17^th^ of December 2022. A cross-sectional study was used to document EIH among patients with intracranial hematomas, as well as the associated risk factors for TBI and EIH development. A prospective study was used to assess the postsurgical patient outcomes including complications, quality of life, and functional outcomes using the Glasgow Outcome Scale (GOS) at 1, 30, 90, and 180 days of observation.

### Study settings

The study was conducted at the Accident and Emergency Department, the operative theatres, and the neurosurgery department at the MNRH in Kampala, Uganda. MNRH, the largest public hospital in Uganda, serves 75% of injured victims in Kampala and is located around 5 km from the city center. The neurosurgery department serves the Kampala city population and neighboring districts, as well as patients referred from all other regions of the country. Monthly, the Accident and Emergency Department attends to 64 urgent surgical evacuations of intracerebral hemorrhage in patients with acute TBI at MNRH. TBI patients were recruited on admission at the Accident and Emergency Department, and then followed up in the operative theatres and the neurosurgery department.

### Study population

All TBI patients, 18 years or older, who presented at the Accident and Emergency department at the MNRH within the period of the study were considered using general screening criteria. Those who had a CT scan with an intracranial bleed were eligible to participate in the study.

### Study participant selection criteria

#### Inclusion criteria

We included traumatic brain injury participants who were; i) aged 18yrs and above with confirmed expansive intracranial hematoma based on two CT scans, ii) post -resuscitation Glasgow Coma Scale (GCS) score of 4 to 14. Patients needed to be eligible for cranial surgery and enrolled in the study within 24 h of initial presentation to the hospital. The remaining intracranial hematomas patients, who did not develop any EIH were identified as the comparison cohort, or control group (No EIH). A written signed informed consent form was obtained from the patient or their next of kin.

#### Exclusion criteria

Patients with (1) known pre-thrombocytopenia; (2) a history of coagulation disorders; (3) used anticoagulants; (4) pregnancy; and (5) the inability to consent before surgical intervention; and (6) who had surgery from the results of the first Ct scan image without waiting for the second were excluded from the study.

### Sampling procedure

Purposive sampling was used to recruit participants. Using the Qualtrics Sample Size Calculator, the calculated sample size of participants was 324 (https://epitools.ausvet.com.au/samplesize). The purposive sampling method was used to recruit participants.

### Study procedures and variables

Patients were recruited from the Accident and Emergency Department and were followed through the operative theatres, postoperatively in the neurosurgery ward, and the neurosurgical outpatient clinics for up to 6 months using a well-designed tool that was incorporated in Redcap software, for the occurrence of complications and recording of GOS and quality of life (QoL). Trained research assistants used the Research Electronic Data Capture (REDCap) system to obtain pertinent demographic, clinical, laboratory, and radiological information from the study participation. Patient demographics were recorded on a case report form (CRF) which included information regarding age, gender, occupation, residence type, geographic regions, and matrimonial state. Outcomes data were obtained at enrollment (baseline) and then at 1 day, 30, 90, and 180- days post discharge. Clinical outcomes were also recorded during these outpatient visits. Data was captured on the specific case-record forms, and outcomes were adjudicated centrally with the use of an outcomes document with case definitions as a guide (details of protocol are provided in the Supplementary Appendix). The principal outcomes that were captured included death due to any of the following complications: imaging evidence of epidural, subdural, contusion, intracerebral hematomas, subarachnoid hemorrhage, clinically apparent spasticity, cerebrospinal fluid leakage, bleeding diastasis, laboratory apparent platelet dysfunction, clinical evidence of fever, infection source gastrointestinal tract (GIT), upper respiratory tract (URT) pneumonia, urinary tract, perinephric, skin abscesses, and wound complication. GOS was used to evaluate the functional outcome following acute brain injury in patients, indicating patients’ level of independence at home and in the community, as well as their capacity to work, engage in social and recreational activities, maintain personal connections, and resume their typical activities following an injury [25, 26]. The Quality of Life after Brain Injury (QoLIBRI) scores were scored on a scale of 0 up to 100, where 100 is the best possible quality of life after traumatic expansive hematoma and 0 is the worst possible quality of life after traumatic expansive hematomas [27].

### Statistical data analysis

Data was captured into the REDCap secured system of the Uganda Cancer Institute (UCI). Non-parametric continuous data was summarized using the median and interquartile range, whereas continuous parametric data was presented using the mean and standard deviation. Categorical variables were expressed as frequencies and percentages. To assess for the risk factors of expansive hematoma, the chi-square test was used along with the univariate analysis to assess for variables that had an independent association with the outcome. Variables with a *p* < 0.2 were considered for multivariate analysis. Modified Poisson regression was used for the multivariate analysis and prevalence ratios and the corresponding 95% confidence interval was calculated for each variable. All variables were evaluated for collinearity and independent relationships. Variables that had *p* < 0.05 were retained in the model and were assessed for dependency using the chunk test. The confounding variables were controlled for using a backward stepwise analysis with an assumed odds ratio difference of more than 10%. Variables with *p* < 0.05 were retained in the model and considered to be statistically significant as risk factors attributable to expansive hematoma. To assess the effect of surgical evacuation on mortality, Kaplan Meier survival curves were used and a log rank test was used to assess for statistical significance. Univariate and Multivariate cox regression analyses were performed to evaluate the predictors of mortality.

### Ethical considerations

The study was approved by the Makerere University School of Medicine Research Ethics Committee and recorded as Mak_SOMREC-2020–38.

## Results

### Study flow of the participants

**Fig. 1:**
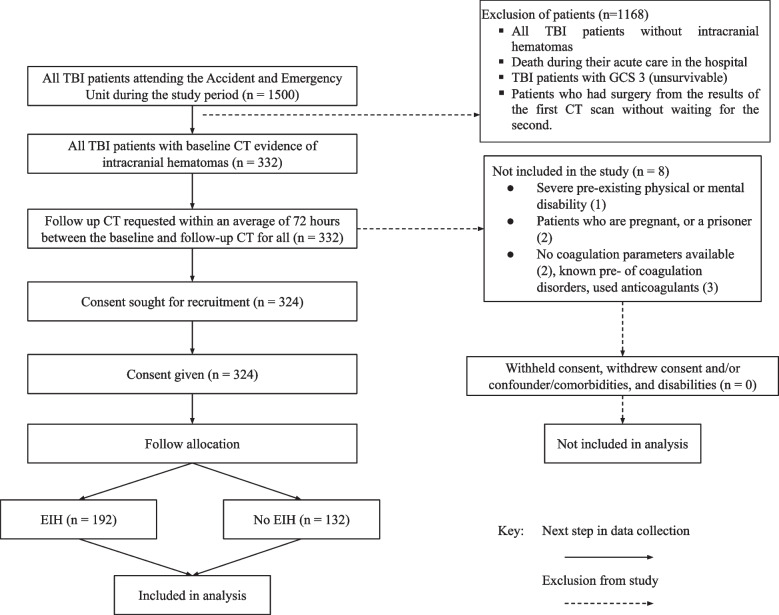
Recruitment flow chart

During the study period, a total of 1500 patients were eligible for enrollment (Fig. 1). Out of these, 21.6% (*n* = 324) were enrolled in the study. In a cohort of 324 patients with intracranial hematomas, 59.3% (*n* = 192) had expansive intracranial hematomas identified on CT scan (Fig. 1).

### Sociodemographic characteristics and injury factors of patients with EIH

The finding from the study showed that most of the patients were male 261 (80.6%) and the majority 152 (48.0%) were motorcyclists known as boda riders (Table 1). The mean age of the study participants was 37.5 ± 17.4 years.

**Table 1:** Baseline demographic and clinical characteristics

**Variables**	**No Expansive intracranial hematoma**	**Expansive intracranial hematoma**	**Total**	***P*- Value**
	**No. (%)**	**No. (%)**	**No. (%)**	
**n (%)**	132 (40.7)	192 (59.3)	324 (100.0)	
**GCS: Eye-opening, n (%)**				
None	17 (12.9)	28 (14.6)	45 (13.9)	
To pain	21 (15.9)	21 (10.9)	42 (13.0)	
To speech	18 (13.6)	18 (9.4)	36 (11.1)	
Spontaneously	76 (57.6)	125 (65.1)	201 (62.0)	0.302
**GCS: Verbal response, n (%)**				
None	17 (12.9)	26 (13.5)	43 (13.3)	
Incomprehensive	19 (14.4)	38 (19.8)	57 (17.6)	
Inappropriate	17 (12.9)	11 (5.7)	28 (8.6)	
Confused	78 (59.1)	115 (59.9)	193 (59.6)	
Oriented	1 (0.8)	2 (1.0)	3 (0.9)	0.197
**GCS: Motor, n (%)**				
None	1 (0.8)	6 (3.1)	7 (2.2)	
Extension to pain	3 (2.3)	5 (2.6)	8 (2.5)	
Flexion to pain	9 (6.8)	14 (7.3)	23 (7.1)	
Withdraws from pain	9 (6.8)	18 (9.4)	27 (8.3)	
Localises to pain	33 (25.0)	30 (15.6)	63 (19.4)	
Obeys commands	77 (58.3)	119 (62.0)	196 (60.5)	0.268
Severity of GCS, mean (sd)	11.6 (3.0)	11.5 (3.4)	11.6 (3.2)	0.791
Severity of GCS, median (iqr)	14.0 (5.0)	14.0 (4.8)	14.0 (5.0)	0.744
Severity of GCS, median (iqi)	14.0 (9.0; 14.0)	14.0 (9.3; 14.0)	14.0 (9.0; 14.0)	0.744
**Severity group, n (%)**				
Mild	78 (59.1)	114 (59.4)	192 (59.3)	
Moderate	28 (21.2)	35 (18.2)	63 (19.4)	
Severe	26 (19.7)	43 (22.4)	69 (21.3)	0.731
**Does patient have unequal pupil size n (%)**				
No	113 (85.6)	166 (86.5)	279 (86.1)	
Yes	19 (14.4)	26 (13.5)	45 (13.9)	0.827
**Pupillary light reflex, n (%)**				
Both Unresponsive	5 (3.8)	8 (4.2)	13 (4.0)	
One Unresponsive	19 (14.4)	24 (12.5)	43 (13.3)	
Both Responsive	108 (81.8)	160 (83.3)	268 (82.7)	0.878
**Are the pupils dilated? (>4mm)?, n (%)**				
Both Dilated	9 (6.8)	14 (7.3)	23 (7.1)	
One Dilated	24 (18.2)	26 (13.5)	50 (15.4)	
Both Normal Size	99 (75.0)	152 (79.2)	251 (77.5)	0.524
**Are the pupils miotic?, n (%)**				
(< 3mm) Bilaterally miotic	7 (5.3)	9 (4.7)	16 (4.9)	
Unilaterally miotic with intact light response	125 (94.7)	183 (95.3)	308 (95.1)	0.802

### Proportion of traumatic brain injury patients presenting with expansive hematomas

**Fig. 2:**
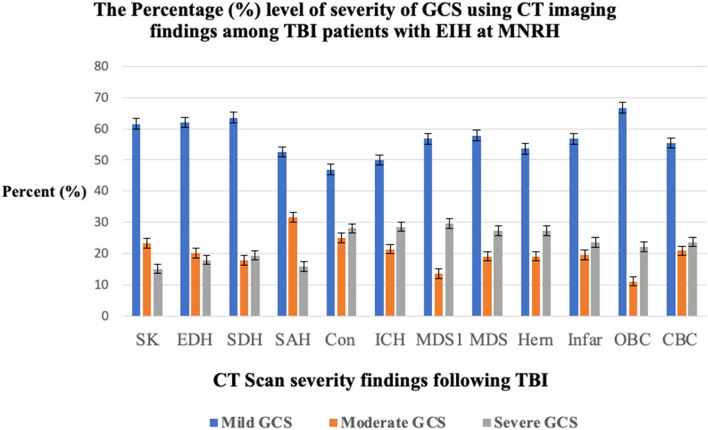
The proportion of traumatic brain injury patients presenting with expansive hematomas (Abbreviations defined in the list of abbreviations)

The finding of the study showed that of the 324 patients with intracranial hematomas, 59.3% (*n* = 192) had expansive hematomas identified on CT scan resulting in a proportion of 0.59 (95% CI: 0.54 to 0.65). Majority of the patients had mild TBI following neurological assessment and imaging findings using GCS (Fig. 2 and Table 2).

**Table 2:** Neurological assessment of all TBI including both patients with and without EIH

**Variables**	**Expansive intracranial hematoma**	**No expansive intracranial hematoma**	**Total**	***P*- Value**
	**n (%)**	**n (%)**	**n (%)**	
*Traumatic brain injury patients (TBI) presented to Accident & Emergency Unit*	192 (59.3)	132 (40.7)	324 (100.0)	
***Age (Years)***				
18-28	64 (33.3)	77 (58.3)	141 (43.5)	
29-38	24 (12.5)	29 (22.0)	53 (16.4)	
39-48	30 (15.7)	9 (6.8)	39 (12.0)	
>48	74 (38.5)	17 (12.9)	91 (28.1)	< 0.001
***Gender***				
Female	39 (20.3)	24 (18.2)	63 (19.4)	
Male	153 (79.7)	108 (81.8)	261 (80.6)	0.634
***Patients’ occupation***				
Farming	26 (14.0)	11 (8.3)	37 (11.7)	
Family business	38 (20.4)	16 (12.1)	54 (17.0)	
Employed	13 (7.0)	9 (6.9)	22 (6.9)	
Boda boda rider	80 (43.0)	72 (54.5)	152 (48.0)	
Taxi driver	29 (15.6)	23 (17.4)	52 (16.4)	0.109
***Residence type / location***				
Rural	106 (55.2)	78 (59.1)	184 (56.8)	
Urban	86 (44.8)	54 (40.9)	140 (43.2)	0.488
***Matrimonial state***				
Unmarried	65 (33.9)	62 (47.0)	127 (39.2)	
Married	127 (66.1)	70 (53.0)	197 (60.8)	0.017
***Comorbidities***				
Hypertension n (%)	27 (14.1)	9 (6.8)	36 (11.1)	
No n (%)	165 (85.9)	123 (93.2)	288 (88.9)	0.041
***Patient with history of alcohol and substances of abuse***				
None	146 (76.0)	109 (82.6)	255 (78.7)	
Smoking	9 (4.7)	1 (0.8)	10 (3.1)	0.044
Alcohol abuse	37 (19.3)	22 (16.6)	59 (18.2)	0.551
***Pre-injury ASA - PS (America Society of anaesthesia - Performance Status)***				
1. A normal healthy patient	155 (80.7)	127 (96.2)	282 (87.0)	
2. A patient with mild systemic disease	15 (7.8)	4 (3.0)	19 (5.9)	
3. A patient with severe systemic disease	22 (11.5)	1 (0.8)	23 (7.1)	<0.001
***Post-injury ASA (American Society of anaesthesia***				
1. A normal healthy patient	146 (76.0)	100 (75.8)	246 (75.9)	
2. A patient with mild systemic disease	17 (8.9)	7 (5.3)	24 (7.4)	
3. A patient with a severe systemic disease	29 (15.1)	25 (18.9)	54 (16.7)	0.364
***HB <11.5g/dL***				
No	18 (9.5)	26 (19.7)	44 (13.7)	
Yes	171 (90.5)	106 (80.3)	277 (86.3)	0.009
***INR >1.2***				
No	79 (42.9)	63 (50.0)	142 (45.8)	
Yes	105 (57.1)	63 (50.0)	168 (54.2)	0.22
***Platelets <100***				
No	24 (12.7)	6 (4.6)	30 (9.4)	
Yes	165 (87.3)	125 (95.4)	290 (90.6)	0.014
***Na+ concentration >145 mEq/L***				
No	34 (18.0)	14 (10.7)	48 (15.0)	
Yes	155 (82.0)	117 (89.3)	272 (75.0)	0.072
***Creatinine >1.2***				
No	105 (55.9)	52 (41.9)	156 (50.2)	
Yes	83 (44.1)	72 (58.1)	155 (49.8)	0.013
***MAP >100mmHg***				
No	118 (62.4)	95 (72.0)	213 (66.4)	
Yes	71 (37.6)	37 (28.0)	108 (33.6)	0.075
* aPTT>18.5*				
No	161 (87.5)	117 (92.9)	278 (89.7)	
Yes	23 (12.5)	9 (7.1)	32 (10.3)	0.128
***AST/SGOT >30 IU/L***				
No	100 (54.3)	56 (46.7)	156 (51.3)	
Yes	84 (45.7)	64 (53.3)	148 (48.7)	0.19
***GGT >30 IU/L***				
No	80 (44.2)	63 (53.8)	143 (48.0)	
Yes	101 (55.8)	54 (46.2)	155 (52.0)	0.104
*ALT >30 IU/L*				
No	112 (60.9)	59 (49.6)	171 (56.4)	
Yes	72 (39.1)	60 (50.4)	72 (43.6)	0.053
***Urea >40***				
No	162 (90.0)	106 (88.3)	268 (89.3)	
Yes	18 (10.0)	14 (11.7)	32 (10.7)	0.647
***SBP on admission >120mmHg***				
No	54 (28.6)	56 (42.4)	110 (34.3)	
Yes	132 (71.4)	76 (57.6)	211 (65.7)	0.01

### TBI Injury risk factors to EIH development and clinical presentations (univariate analysis)

The findings from the study showed that clinically, only 11.1% (*n* = 36) participants had hypertension, and 18.2% (*n* = 59) had a history of alcohol intake (Table 1). Comparison of patients with expansive hematomas to those without, the findings showed that the prevalence of comorbidities was statistically significant (*P* = 0.041), smoking habits (*P* = 0.044), matrimonial state (*P* = 0.017), and age (*P* < 0.001). There were no differences identified between female and male patients, occupation, type of residence, and alcohol abuse (*P* > 0.05) (Table 1). In addition, 13.0% (*n* = 42) of the patients had systemic disease, 5.9% (*n* = 19) had mild and 7.1% (*n* = 23) had severe disease as assessed by the America Society of Anesthesia (ASA) performance status (Table 1). EIH patients with systemic disease differed significantly when compared to those without expansive intracranial hematomas. There were no differences in post-injury ASA between the two groups (Table 1). Similarly, there were differences in the two groups regarding the clinical and laboratory assessment of the participants, HB < 11.5 g/dl, a platelet count of < 100, sodium levels of more than 145, creatinine levels of more than 1.2, MAP > 100 mmHg, ALT and SBP on admission > 120 mmHg. There were no differences between the two groups regarding the INR, AST, GGT, urea, and aPTT (Table 1).

### Risk factors leading to expansive intracranial hematoma development following TBI (multivariate analysis)

All variables with a *p*-value less than 0.2 were included in the multivariate analysis. Age, patients’ occupation, mechanism of injury, comorbidities, smoking, matrimonial state, pre-injury ASA, GCS Verbal response, HB, platelets, sodium, creatinine, mean arterial blood pressure, AST, ALT, GGT, systolic blood pressure on admission, injury on the head and skull, epidural hematoma, subdural hematoma, contusion, intracerebral hemorrhage, midline shift, presence of herniation, presence of infarction, surgical treatment, type of surgery, and aPTT had levels of significance of less than 0.2. These were considered to have independent associations with expansive hematomas and were therefore included in the multivariate analysis. The multivariate analysis revealed that participants 48 years and older PR = 1.56 (95% CI: 1.23 to 1.98; *P* < 0.001), age 39 to 48 PR = 1.54 (95% CI: 1.20 to 1.97; *P* = 0.001), smoking PR = 1.21 (95% CI: 1.00 to 1.47; *P* = 0.048), those who have a severe systemic disease PR = 1.36 (95% CI: 1.14 to 1.64; *P* = 0.001), and those with the presence of infarction PR = 2.26 (95% CI: 1.29 to 3.95; *P* = 0.004) were found to be at greater risk for expansive hematomas among patients with traumatic brain injuries (Table 3).

**Table 3:** Multivariate analysis of sociodemographic and clinical characteristics of patients with Expansive Intracranial Hematoma

**Variables**	**Patients with Expansive Intracranial Hematomas**	**Prevalence Ratio (PR)**	**95% CI**	***P*- Value**
	**n (%)**			
***Age (Years)***				
18-28	*141 (43.5)*	*Ref*		
29-38	53 (16.3)	0.97	0.69 to 1.36	0.852
39-48	39 (12.0)	1.54	1.20 to 1.97	0.001
>48 (91)	91 (28.1)	1.56	1.23 to 1.98	< 0.001
**Patient with history of toxic substances**				
None	*109 (33.6)*	*Ref*		
Smoking (10)	10 (3.1)	1.21	1.00 to 1.47	0.048
**Pre-injury ASA - PS (American Society of Anesthesia - Performance Status) **				
Normal healthy patient	*282 (87.0)*	*Ref*		
Patient with mild systemic disease	19 (5.8)	1.08	0.850 to 1.38	0.514
Patient with severe systemic disease	23 (7.1)	1.36	1.14 to 1.64	0.001
**Presence of swirl sign, n (%)**				
No	*32 (9.8)*	*Ref*		
Yes	292 (90.1)	2.26	1.29 to 3.95	0.004

### Treatment modalities, evolution, type, timing of surgery, complications, and baseline quality of life

The findings from the study showed that there were differences between the two groups in regards to the types of surgical evacuation (p ≤ 0.001) and site of surgery (*p* = 0.045) (Table 4). The findings further showed that 95.1% (n = 308) of IH patients who underwent surgery for hematoma evacuation, 89.5% (*n* = 290) underwent craniotomy and 5.6% (*n* = 18) underwent decompressive craniectomy, and 4.9% (*n* = 16) were managed conservatively. About 46.2% (*n* = 141) patients presented late at the Accident & Emergency unit and therefore they had delayed surgical evacuation, 36.7% (*n* = 112) had late and 17.0% (*n* = 52) underwent early surgery depending on the time of patient’s presentation at the unit. The findings further showed that 43.2% (*n* = 140) of patients developed EIH over 72 h post-injury and most of them, 23.5% (*n* = 63) underwent frontal cranial surgery for hematoma evacuation (Table 4). The findings on the overall health-related quality of life of TBI patients with intracranial hematomas who underwent surgical evacuation improved with time. However, the findings on the health-related quality of life among patients with EIH and those without, showed that the quality of life was lower among patients with EIH over a 6-month post-surgical evacuation follow up (Quality of Life after Brain Injury score: 88.0(8.6) vs 85.0(9.5), *P* = 0.010) (Table 4).

**Table 4:** Treatment modalities, evolution, type, timing of surgery, complications, and baseline quality of life

**Variables**	**No Expansive intracranial hematoma**	**Expansive intracranial hematoma**	**Total**	***P*- Value**
	**No. (%)**	** No. (%)**	**No. (%)**	
**n (%)**	132 (40.7)	192 (59.3)	324 (100.0)	
**Evolution of the EIH, n (%)Evolution of the EIH, n (%)**				
< 24hs Urgent	23 (17.4)	33 (17.2)	56 (17.3)	
>24hs< 72hs Late	54 (40.9)	74 (38.5)	128 (39.5)	
>72hs Delay	55 (41.7)	85 (44.3)	140 (43.2)	0.889
**Timing of decompression, n (%)**				
Late	48 (39.0)	64 (35.2)	112 (36.7)	
Early	25 (20.3)	27 (14.8)	52 (17.0)	
Delay	50 (40.7)	91 (50.0)	141 (46.2)	0.225
**Type of surgery, n (%)**				
Evacuation only	19 (15.6)	71 (39.4)	90 (29.8)	
Decompression only	2 (1.6)	1 (0.6)	3 (1.0)	
Both craniotomy	96 (78.7)	93 (51.7)	189 (62.6)	
Both craniectomy	5 (4.1)	15 (8.3)	20 (6.6)	< 0.001
**Intraoperative complications, n (%)**				
No	53 (40.2)	83 (43.2)	136 (42.0)	
Yes	79 (59.8)	109 (56.8)	188 (58.0)	0.581
**Baseline quality of life**				
QOL Overall score total 1 day, mean (sd)	50.0 (15.3)	49.0 (16.8)	49.4 (16.2)	0.596
QOL Overall score total 30 days, median (iqr)	61.6 (19.6)	62.2 (17.2)	61.9 (18.2)	0.771
QOL Overall score total 90 days, median (iqr)	79.4 (9.5)	76.3 (10.4)	77.5 (10.1)	0.011
QOL Overall score total 180 days, median (iqr)	88.0 (8.6)	85.0 (9.5)	86.0 (9.3)	0.01

### Postoperative Clinical Outcomes of intracranial hematomas patients following TBI

The findings from the study showed that the respective inpatient postoperative risks were 10.2% for death, 58.0% for intraoperative complications, 15.7% for early posttraumatic seizures (PTS), 11.7% for coma, 11.7% for brain oedema, 4.9% for wound infection, 4% for nutrition deficit, 3.4% for pneumonia, 2.8% for CSF leakage, 1.2% for subdural hygroma (Table 5). However, about 88.4% (*n* = 282) IH patients remained alive until the end of the study period. Among these patients, 73.8% (*n* = 208) were discharged with favourable conditions (defined by GOS of > 3) and 26.2% (*n* = 74) were discharged with unfavourable conditions (defined by GOS of < 3) (Table 5).

**Table 5:** Baseline complications occurrence for adult traumatic brain injury patients with intracranial hematomas

**Complications**	**No EIH**	**EIH**	**Total**	***P*-Value**
**n (%)**	**132 (40.7)**	**192 (59.3)**	**324 (100.0)**	
** No**	53 (40.2)	83 (43.2)	136 (42.0)	
** Yes**	79 (59.8)	109 (56.8)	188 (58.0)	0.581
Infection (Y), n (%)	8 (6.1)	8 (4.2)	16 (4.9)	0.439
Pneumonia (infection of the lungs) (Y), n (%)	8 (6.1)	3 (1.6)	11 (3.4)	0.028
Early PTS (Y), n (%)	22 (16.7)	29 (15.1)	51 (15.7)	0.704
Brain swelling (Y), n (%)	11 (8.3)	27 (14.1)	38 (11.7)	0.115
Leakage of cerebrospinal fluid) (Y), n (%)	4 (3.0)	5 (2.6)	9 (2.8)	0.816
Subdural hygroma	3 (2.3)	1 (0.5)	4 (1.2)	0.161
Nutrition deficit	5 (3.8)	8 (4.2)	13 (4.0)	0.864
Coma (Y), n (%)	13 (9.8)	25 (13.0)	38 (11.7)	0.383
Death	16 (12.1)	17 (8.9)	33 (10.2)	
Alive	116 (87.9)	175 (91.1)	282 (88.4)	0.073

### Overall Kaplan Meier 16 months mortality of intracranial hematomas patients following TBI

**Fig. 3:**
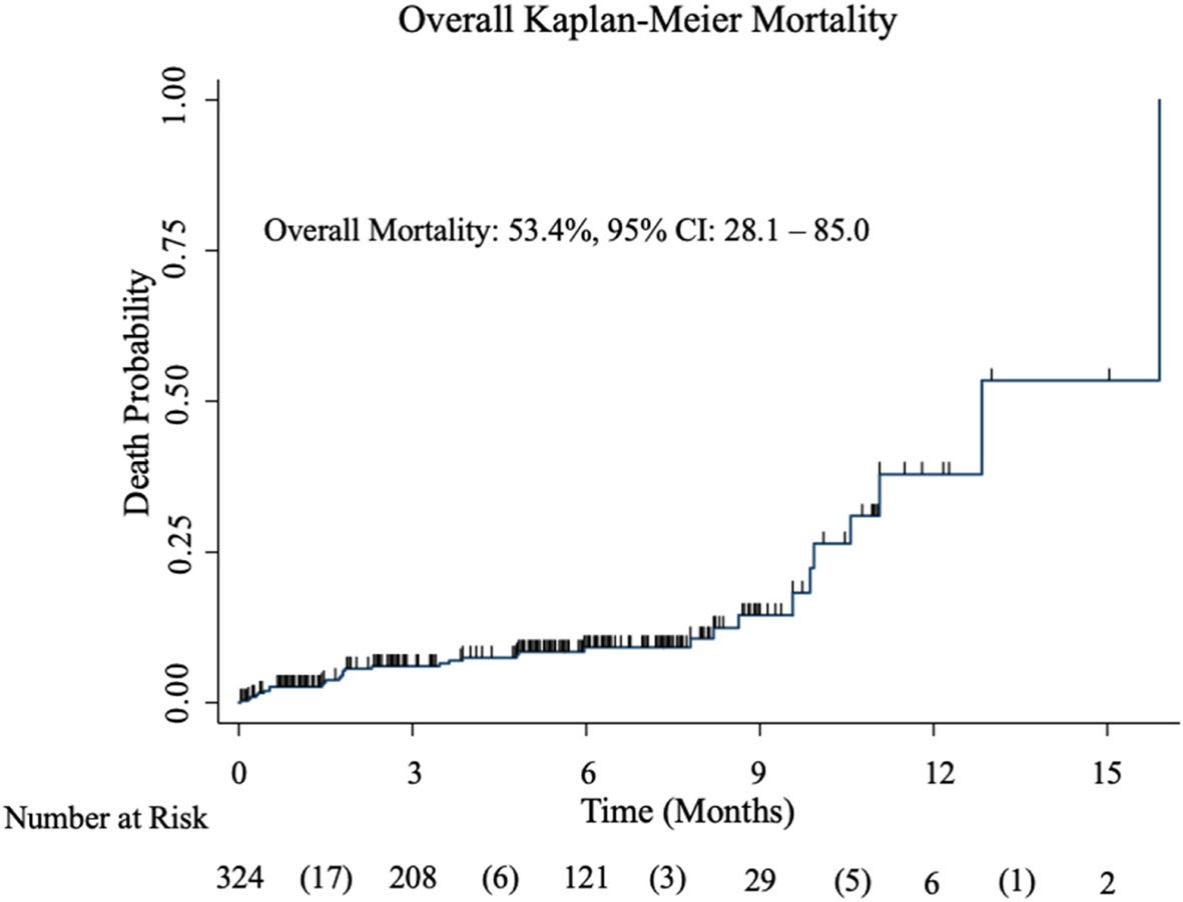
Overall Kaplan Meier 16 Months mortality

The findings from the study showed that the overall 16 months mortality of patients with expansive intracranial hematoma after surgery increased over time (Fig. 3). The Kaplan Meier 16 months mortality was 53.4%, 95% CI = (28.1 to 85.0) (Fig. 3). The Kaplan–Meier curve showed increased mortality between the 5th and 8th month after traumatic brain injury (TBI), indicating a critical period during which individuals are at higher risk of death (Fig. 3).

### Predictors of mortality of intracranial hematomas patients following TBI

All variables with a *p*-value less than 0.2 in the univariate analysis were included in the multivariate analysis. Age, mean arterial pressure, GCS, GOS, complications, quality of life, and brain hematomas subgroups had levels of significance of less than 0.2. These were considered to have independent associations with mortality among TBI patients with brain hematomas and were therefore included in the multivariate analysis.

The findings from the study, the multivariable Cox regression analysis showed that participants between the ages of 31 and 45 were 34.1 times more likely to be vulnerable to death when compared to patients aged 18 to 30 years with adjusted hazard ratio (AHR = 34.1; 95% CI: 0.03–3.3). Similarly, patients between the ages of 46 and 60 were 6.5 times more likely subject to death compared with patients aged between 18 to 30 years (AHR = 6.5; 95% CI: 0.09–5.03). Participants aged 61 and above were 0.9 times more likely to die compared to patients aged 18 to 30 years (AHR = 0.9; 95% CI: 0.002–39). The hazard of death among those who had an elevated MAP of 95mmHG and above was 5.4 times higher compared to those with MAP less than 95 mmHg (AHR = 5.4; 95% CI: 0.04–6.5). The hazard of death among patients with a baseline GCS score at admission ≤ 8 was 2.4 times higher compared to patients with a GCS score ≥ 13 (AHR = 2.4; 95% CI: 4.8–12.05). Patients with a baseline GCS score at admission of 9–12 had a 6.6 times greater risk of death when compared to patients who had a GCS score of 13–15 (AHR = 6.6; 95% CI: 4.6–9.6). Patients with favorable outcomes had a 3.04 times lower risk of death compared to those with unfavorable outcomes based on GOS (AHR: 3.04; 95% CI: 4.5–20.2) (Table 6).

**Table 6:** Predictors of mortality in all TBI including both EIH and no EIH patients

**Variables**	**Death**	**Alive**	**Bivariate CHR (95% CI)**	**Multivariate AHR (95% CI)**
	**No. (%)**	** No. (%)**		
n (%)	33 (10.2)	291 (89.8)		
**Age group in years, n (%)**				
18 to 30	13 (39.4)	145 (49.8)	1	1
31 to 45	9 (27.3)	52 (17.9)	2.0 (0.8-4.7)	34.1 (0.03 - 3.3)
46 to 60	8 (24.2)	57 (19.6)	2.02 (08-4.9)	6.5 (0.09 - 5.03)
61 and above	3 (9.1)	37 (12.7)	0.7 (0.1-3.5)	0.9 (0.002 - 39)
**Mean arterial pressure**				
Less than 95			1	1
95 and above			2.2 (1.08 - 4.6)	5.4 (0.04 - 6.5)
**Severity of GCS**				
Mild	7 (21.2)	185 (63.9)	1	1
Moderate	5 (15.2)	58 (19.9)	2.2 (0.7 - 7.01)	6.6 (4.6 - 9.6)
Severe	21 (63.6)	48 (16.5)	9.3 (3.9 - 22.09)	2.4 (4.8 - 12.05)
**GOS**				
GOS Unfavourable	26 (78.8)	59 (20.3)	1	1
GOS Favourable	7 (21.2)	232 (79.7)	12.2 (5.2 - 28.4)	3.04(4.5-20.2)
** Complication**				
Fever (Y), n (%)	18 (54.5)	15 (5.2)	9.1 (4.4 - 18.7)	0.03 (0.0007 - 1.6)
Infection (Y), n (%)	25 (75.8)	32 (11.0)	15.7 (7.05 - 35.3)	2.1 (0.0009 - 4.8)
Spasticity (Y), n (%)	12 (36.4)	59 (20.3)	2.2 (1.09 - 4.7))	9.8 (0.06 - 1.5)
Bleeding diathesis (Y), n (%)	21 (63.6)	79 (27.1)	5.2 (2.5 - 11.02)	4.9 (1.5 - 15.2)
Wound dehiscence (Y), n (%)	2 (6.1)	3 (1.0)	5.08 (1.1 - 21.8)	7.3 (0.2 - 19.5)
CSF Leak (Y), n (%)	4 (12.1)	5 (1.7)	2.4 (0.8 - 7.4)	1.6 (0 - 0)
Nutrition deficit (Y), n (%)	3 (9.1)	10 (3.4)	2.5 (0.7 - 8.4)	0.00003 (5.5 - 20.3)
**QOLIBRI at 1 day, n (%)**				
0 to 49	23 (69.7)	136 (46.7)	1	
50 to 100	10 (30.3)	155 (53.3)	0.5 (0.2 - 1.1)	4 (0.003 - 4.1)
**Epidural hematoma, n (%)**				
No	17 (51.5)	128 (44.0)	1	
Yes	16 (48.5)	163 (56.0)	0.9 (0.4-1.9)	0.3 (0.01 - 7.2)
**Subdural Hematoma, n (%)**				
No	26 (78.8)	133 (45.7)	1	
Yes	7 (21.2)	158 (54.3)	0.3(0.1 - 0.9)	1.9 (0 - 0)
**Subarachnoid Hemorrhage (SAH), n (%)**				
No	28 (84.8)	277 (95.2)	1	
Yes	5 (15.2)	14 (4.8)	4.4 (1.7 - 11.8)	0.2 (0.001 - 5.3)
**Contusion(s), n (%)**				
No	27 (81.8)	265 (91.1)	1	
Yes	6 (18.2)	26 (8.9)	3.3 (1.3 - 8.2)	7.01(0 - 0)
**Expansive Hematoma, n (%)**				
No	20 (60.6)	112 (38.5)	1.9 (0.7 - 3.2)	3.8(0 - 0)
Yes	13 (39.4)	179 (61.5)	1	

## Discussion

There is a significant deficit of reliable data and research on the extent of the effect of EIH on neurosurgical outcomes, particularly from the prevalence, risk factors, predictors of mortality, QOLIBRI, and functional outcomes perspectives in Uganda and related low-and-middle-income countries (LMIC). Consequently, this study set out to assess the burden, risk factors, surgical evacuation outcomes, and predictor of mortality for EIH following traumatic brain injury (TBI). Through the fitness of our study design, we were able to collect adequate information and data regarding TBI patients, factors contributing to EIH, predictors of mortality, and related surgical outcomes to further inform community-based interventions. This study found that the prevalence of EIH was 59.3% in adult TBI patients and risk factors for EIH for adult TBI patients included increased age above 39 years, smoking, having severe systemic disease, and the presence of swirl sign (Tables 1, 2 and 3).

In addition, the 16 months Kaplan Meier mortality was 53.4% (95% CI = 28.1 to 85.0) and predictors of mortality were age, MAP values above 95 mmHg, low GCS, a complication such infection, spasticity, wound dehiscence, CSF leaks, having GOS < 3, QOLIBRI of less than 50, ASDH, contusion, and EIH (Fig. 3 and Table 6). Furthermore, the Kaplan–Meier curve displayed increased mortality between the 5th and 8th month after traumatic brain injury. This indicates a critical period during which individuals are at higher risk of death. There could be several factors contributing to this observation including delayed complications, rehabilitation challenges, and long-term consequences. Some complications or health issues related to TBI may take time to manifest and become more severe. These complications could include infections, neurological deterioration, or secondary injuries that lead to increased mortality during this specific time frame. The 5th to 8th month after TBI is a crucial phase of rehabilitation for many patients. It is a period when individuals may face challenges in their recovery, such as motor and cognitive impairments, emotional and psychological difficulties, or difficulties adapting to daily activities. These challenges can indirectly contribute to an increased risk of mortality. TBI can have long-lasting effects on an individual's overall health and well-being. The 5th to 8th-month post-injury may be a critical time when some individuals experience progressive deterioration in their condition or encounter complications related to TBI, leading to higher mortality rates.

### Sociodemographic characteristics and injury factors of patients with EIH

Demographic characteristics in this study showed that the mean age of the study participants was 37.5 ± 17.4 years. Majority of the participants were male (80.6%). Almost half of the study participants were bodaboda riders(46.9%), the majority of patients were coming from rural areas and 60.8% of patients were married (Table 1). Bodaboda riders are the main contributor of TBI, hence expansive hematoma. These findings agree with previous studies in Uganda and elsewhere [28]. Given the high rate and early time course of this phenomenon, participants of different socio-demographic and other characteristics were assessed for the articulated result. In the univariate model, 43.5% of the participants were between 18 and 28 years. This finding concurs with a study conducted by Maas et al. [1] which revealed that TBI affects the more productive age groups, which can put additional pressure on existing economic and healthcare burdens [1]. In addition, 56.2% of TBI occurred in rural areas in Uganda. This result is consistent with a study conducted by LaGrone et al. [29], which showed that the high incidence of TBI in developing regions may be partly due to an increased number of individuals with a high demand for unsafe movement and partly due to poor infrastructure. Other contributing factors include inadequate enforcement of traffic laws, alcohol abuse, and inefficient response from an already weak healthcare system [29].

According to this study, the mean age (SD) of patients with EIH was statistically different from that of patients without EIH (42.3 ± 17.9 vs. 30.5 ± 14.0 years, *p* = 0.000). It is consistently demonstrated that increased age is associated with intracranial hematoma progression and age-related structural deficiencies in the microvasculature, endothelial loss, and lower resting CBF making people more susceptible to developing EIH [2, 13, 30, 31].

### The proportion of traumatic brain injury patients presenting with expansive hematomas

The prevalence of EIH among TBI patients presenting at the Accident and emergency unit at MNRH was 59.3% among adult TBI patients. These findings were consistent with previous studies which showed that the rate of EIH following TBI ranged from 38 to 59% of cerebral hemorrhages [6, 11, 32–34], but was lower than a study by Adatia and colleagues who reported it to be as high as 75% [13]. These variations may, in part, have resulted from the absence of a common definition of EH in the literature [35–37]. The timing between baseline (first scan at the initial presentation at the Accident and emergency unit) and follow-up scans, research inclusion criteria, and various hematoma volume measuring techniques, on the other hand, could all contribute to this variation in proportion among studies [13]. In addition, lack of standardized protocol on EIH case definition and hence timely management of the case often leads to unfavorable outcomes and poor survival rate long term, therefore there is need to continuously monitor TBI patients with intracranial hematoma with evidence on initial CT scan for any progression of EIH and thus instituting appropriate case management plan including surgical evacuation. From the findings, a protocol could be put in place for risk stratifying those patients to determine how large of an EIH would lead to specific surgical or medical interventions. The study also determined that among TBI patients admitted to MNRH, following 2 CT scans with evidence of increased hematoma volume of over 33% or absolute hematoma growth over 6 mL from the initial scan, EIH was common in all subgroups of intracranial hematomas. The likelihood of EIH for a given lesion was 51.5% for EDH, 51.1 for SDH, 47.6% for ICH, 37.5% for contusions, and 40% for SAH. This result correlates with reports from several series, which demonstrated that the rate of EIH after TBI ranged from 38 to 59% of intracranial hemorrhages [6, 11, 32–34], but presented a lower prevalence compared to results from a study conducted by Adatia and colleagues, who indicated the rate of EIH to be 75% [13]. These differences, in part, may have been due to a lack of standardized definitions of EIH across the literature [35–37]. However, different methods of hematoma volume assessment, study inclusion criteria, and timing between baseline and follow-up scans may explain this discrepancy in proportion across studies [13]. In addition, the findings from the study showed that intracranial hematomas enlarged over time and there were 43.2% of patients developed EIH over 72 h. The findings differ from the findings of the study conducted by Adena et al.(2012), which reported that over 75% of intracranial bleeding occurred within 2 h from the time of the initial head CT scan [4] (Table 4). Therefore, there is variation in the time of EIH occurrence on the initial first CT scan.

### Risk factors leading to expansive intracranial hematoma development following TBI

According to this study, TBI patients aged 48 years and above were 1.56 times more likely to be at risk for EIH than their counterparts. Similarly, TBI patients aged between 39 to 48 years were 1.54 times more likely to be at risk for EIH than TBI patients in other age groups, which is consistent with previous studies [8, 31, 36, 38]. In addition, the prevalence risk of developing EIH in adult TBI patients was 1.21 times more likely in patients with smoking behavior which is lower compared with a study conducted in Korea (sixfold) [39]. The possible explanation for increased likelihood of EIH in smokers may be due to reduced cerebral blood flow within the penumbral zone and greater fragility of vessels [13, 40].

In the present study, TBI patients with severe systemic disease, on the other hand, were 1.36 times more likely to develop EIH than their counterparts. This finding aligns with previous studies where severe systemic diseases like hypertension and diabetes were associated with EIH [5]. Systolic blood pressure (SBP) is correlated with EIH [5, 41], and patients with post-admission SBP more than 160 mmHg are at a considerably higher risk for expanding hematomas [41, 42]. This may be partially explained by the ongoing rupture and bleeding of small blood vessels, making early blood pressure a potential therapy target. This study found that having a background of hypertension was positively related to hematoma enlargement. In addition, patients with a history of hypertension are four times more likely to experience intracranial hematoma expansion than those without a past of hypertension. Endovascular malfunction and cerebrovascular remodeling were seen among patients with chronic hypertension. These changes may be associated with raised blood–brain barrier permeability [13, 43].

Lastly, TBI patients with the presence of a swirl sign have a 2.26-times higher risk of developing an EIH when compared to patients who had no swirl sign. This result was supported by a recent meta-analysis study which revealed that swirl sign has a high specificity for predicting EIH with a pooled positive likelihood ratio of 2.2 (95%CI 1.8–2.5) in intracerebral hemorrhage [44]. In other studies, this imaging marker was associated with EIH and overall poor outcomes [45–50]. This result, however, is inconsistent with research conducted by Boulouis et al., where the Swirl sign was not related to EIH, as revealed by the multivariate analysis [46]. Although our study suggests that the presence of swirl signs is a risk factor for developing EIH, further exploration is required to confirm the role of this factor in EIH prevalence.

### Treatment modalities, evolution, type, timing of surgery, complications, baseline quality of life, and predictors of mortality of intracranial hematoma patients following TBI

In this cohort of EIH patients following TBI, 33 (10.2%) died between the 16^th^ of June 2021 to the 17th of December 2022 with increased age (> 31 years), increased MAP (> 95 mmHg), decreased GCS (< 12), decreased GOS (< 3), decreased QOL (< 50%), SDH, contusions, infection source from GIT, URT, perinephric abscess, fever, platelet dysfunction, skin abscess, wound dehiscence, spasticity, and CSF leakage all contributing to increased risk of death. Older age is generally associated with a higher risk of mortality in various medical conditions, including traumatic brain injuries. Advanced age may lead to reduced physiological reserves and increased vulnerability to severe injuries. Elevated MAP values (> 95 mmHg) can indicate systemic hypertension, which may exacerbate intracranial bleeding and increase the risk of mortality. These findings concur with a study that reported that the survival of TBI patients were low and that elevated blood pressure and nonreactive pupils were predictors of mortality [51]. A lower GCS (< 12) score suggests more severe brain injury and impaired consciousness. Therefore, patients with a decreased GCS were found to be at higher risk of death due to the severity of their brain trauma. Furthermore, the mortality rate among severe TBI (GCS ≤ 8) patients with intracranial hematomas was 63.6%, which is higher compared with a study conducted in Ethiopia where the cumulative incidence of death was 49.71% [51], but slightly lower compared with a prospective study in Uganda where the mortality among severe TBI patients with hyperglycemia at MNRH was 68.8% [52]. A GOS (< 3) score below 3 indicates a poor outcome and increased mortality risk. A low QOL (< 50%) score indicates compromised well-being and may be associated with more severe injuries and an increased risk of death. In addition, the present findings differ from a retrospective study where the mortality rate observed among severe TBI patients was 21.8% [53]. This observed difference in mortality rate can be explained by the sample size, changes in the treatment protocols, and accessibility to intensive care units.

The present study had limitations including, missing values and recall bias on some of the information during the follow-up period. This was expected and the research team increased 10% of possible dropout or missing data. The prospective nature of the study required that patients be followed up for periods including after discharge. This carries with it the risk of loss to follow-up, which has partly been addressed by adjusting the sample size for loss to follow-up. Phone calls were used to help in addressing the loss of follow-up. Patients had CT scans when their neurological condition deteriorated. Even in the absence of neurological decline, changes in serial CT scans may be seen. However, previous studies have not found any abnormalities on serial CT scans in the absence of neurological deterioration that results in therapeutic decision-making. Therefore, the rate of hematoma enlargement may have been underestimated in this analysis. Most of such undetected enlargements, however, are most likely asymptomatic and do not require any therapeutic interventions. Neurological examination was performed by the attending neurosurgeons, senior neurosurgery residents, or the trained research assistants in the Neurosurgery Department. The date, time, and results of the initial and all subsequent scans were recorded. Therefore, interobserver variation can be expected among the different neurological examinations. Nevertheless, this is one of the largest studies to date on EIH, which allows our team to contribute to the broader conversation on TBI in low-resource settings. The current study highlights the burden of EIH, the associated risk factors, the surgical outcomes, and predictors of mortality among TBI patients admitted to the MNRH emergency unit as well as country-wide in Uganda following RTA, assaults, and falls. Future studies must continue to assess (1) the effect of timing on surgery and patient outcomes among adult patients with EIH; (2) survival trends and predictors of mortality among adult patients with EIH; (3) long-term health-related quality of life changes for adult patients in Uganda with or without traumatic EIH.

## Conclusion

Expansive intracranial hematoma is common in low- income countries specifically Uganda with a prevalence of 59.3% of traumatic EIH. Following the recruitment of TBI patients at the Mulago National Referral Hospital, risk factors for EIH were found to include increased age above 39 years, smoking, having severe systemic disease, and presence of swirl sign. The Kaplan Meier mortality was 53.4% (95% CI = 28.1 to 85.0). Old age, MAP values above 95 mmHg, low GCS, complications such as infection, spasticity, wound dehiscence, CSF leaks, having GOS < 3, QoLIBRI < 50, ASDH, and contusion are predictors of mortality for EIH among adult patients with TBI. These elucidated factors can be helpful when screening patients at high risk of hematoma enlargement in future clinical trials and should be investigated when developing a comprehensive predictive score based on demographic, pre and post-heathy American Society of Anesthesia, and non-contrast CT markers in the future. These data can help create informed community-based interventions, as well as contribute to the greater discussion on TBI in low-resource settings.

### Supplementary Information

**Additional file 1.** Appendices.

## Data Availability

Datasets used in the current study are available from the corresponding author upon reasonable request.

## Abbreviations

CBC: Compressed basal cistern
Con: Contusion
EDH: Epidural hematoma
EIH: Expansive intracranial hematoma
GCS: Glasgow coma scale
GOS: Glasgow outcome scale,
Hern: Presence of herniation
HIC: High-income countries
Infar: The presence of infarction
ICH: Intracranial hemorrhage
LMIC: Low-and middle-income countries
MDS1: Midline shift < 1 cm
MDS: Midline shift ≥ 1 cm
MNRH: Mulago National Referral Hospital
OBC: Open basal cistern
PR: Prevalence risk
QoLIBRI: Quality of life after brain Injury
RCT: Randomized clinical trials
TBI: Traumatic Brain Injury
TEH: Traumatic expansive hematoma
SAH: Subarachnoid hemorrhage
SDH: Subdural hematoma
SK: Skull fracture
MAP: Mean arterial pressure
