# Supplementary material for: Burden, risk factors, neurosurgical evacuation outcomes, and predictors of mortality among traumatic brain injury patients with expansive intracranial hematomas in Uganda: a mixed methods study design
Source: BMC Surg. 2023 Oct 25;23:326. doi: 10.1186/s12893-023-02227-9 (PMC10601114; doi:10.1186/s12893-023-02227-9)
Supplement: Supplementary file 1 — Additional file 1. Appendices. [file 12893_2023_2227_MOESM1_ESM.docx]

**Appendices**

**Table 1 : Baseline quality of life patterns**

| **Variables** | **No expansive hematoma**  **No. (%)** | **Expansive hematoma**  **No. (%)** | **Total**  **No. (%)** | **P- Value** |
| --- | --- | --- | --- | --- |
| n (%) | 132 (40.7) | 192 (59.3) | 324 (100.0) |  |
| QOL Overall score total 24 HRs, mean (sd) | 15.0 (4.6) | 14.7 (5.0) | 14.8 (4.9) | 0.600 |
| QOL Overall score total 24 HRs, median (iqr) | 15.0 (6.0) | 15.0 (7.0) | 15.0 (6.0) | 0.568 |
| QOL Overall score total 24 HRs, median (iqi) | 15.0 (12.0; 18.0) | 15.0 (11.0; 18.0) | 15.0 (12.0; 18.0) | 0.568 |
| Overall QOL first 24 hrs, mean (sd) | 50.0 (15.3) | 49.0 (16.8) | 49.4 (16.2) | 0.596 |
| Overall QOL first 24 hrs, median (iqr) | 50.0 (20.0) | 50.0 (23.3) | 50.0 (20.0) | 0.563 |
| Overall QOL first 24 hrs, median (iqi) | 50.0 (40.0; 60.0) | 50.0 (36.7; 60.0) | 50.0 (40.0; 60.0) | 0.563 |
| QOL Overall score total 30 days, mean (sd) | 18.5 (5.8) | 18.6 (5.1) | 18.6 (5.4) | 0.919 |
| QOL Overall score total 30 days, median (iqr) | 19.5 (5.0) | 19.0 (6.0) | 19.0 (5.8) | 0.646 |
| QOL Overall score total 30 days, median (iqi) | 19.5 (17.0; 22.0) | 19.0 (16.0; 22.0) | 19.0 (16.3; 22.0) | 0.646 |
| Overall QOL 30 days, mean (sd) | 61.6 (19.6) | 62.2 (17.2) | 61.9 (18.2) | 0.771 |
| Overall QOL 30 days, mean (sd) | 61.6 (19.6) | 62.2 (17.2) | 61.9 (18.2) | 0.771 |
| Overall QOL 30 days, mean (sd) | 61.6 (19.6) | 62.2 (17.2) | 61.9 (18.2) | 0.771 |
| QOL Overall score total 90 days, mean (sd) | 23.8 (2.8) | 22.9 (3.1) | 23.2 (3.0) | 0.011 |
| QOL Overall score total 90 days, median (iqr) | 24.0 (4.0) | 23.0 (4.0) | 23.0 (3.3) | 0.010 |
| QOL Overall score total 90 days, median (iqi) | 24.0 (22.0; 26.0) | 23.0 (21.0; 25.0) | 23.0 (21.8; 25.0) | 0.010 |
| Overall QOL 90days, mean (sd) | 79.4 (9.5) | 76.3 (10.4) | 77.5 (10.1) | 0.011 |
| Overall QOL 90days, median (iqr) | 80.0 (13.3) | 76.7 (13.3) | 76.7 (10.8) | 0.010 |
| Overall QOL 90days, median (iqi) | 80.0 (73.3; 86.7) | 76.7 (70.0; 83.3) | 76.7 (72.5; 83.3) | 0.010 |
| QOL Overall score total 180 days, mean (sd) | 26.4 (2.6) | 25.5 (2.9) | 25.8 (2.8) | 0.010 |
| QOL Overall score total 180 days, median (iqr) | 27.0 (4.0) | 26.0 (4.0) | 27.0 (4.0) | 0.007 |
| QOL Overall score total 180 days, median (iqi) | 27.0 (24.0; 28.0) | 26.0 (24.0; 28.0) | 27.0 (24.0; 28.0) | 0.007 |
| Overall QOL 180 days, mean (sd) | 88.0 (8.6) | 85.0 (9.5) | 86.0 (9.3) | 0.010 |
| Overall QOL 180 days, median (iqr) | 90.0 (13.3) | 86.7 (13.3) | 90.0 (13.3) | 0.007 |
| Overall QOL 180 days, median (iqi) | 90.0 (80.0; 93.3) | 86.7 (80.0; 93.3) | 90.0 (80.0; 93.3) | 0.007 |

* Comparison of potential quality of life risk factors for 192 patients with and 132 without EH.

Abbreviations: QOL = quality of life;

**Table 3: Mulivariate analysis of sociodemographic and clinical characteristics of patients with expansive hematomas**

| **Variable** | **Unadjusted PR (95% CI) univariate** | **Adjusted PR (95% CI)** | **P-value** |
| --- | --- | --- | --- |
| **Age** |  |  |  |
| 18-28 (141) | ***Ref*** |  |  |
| 29-38 (53) | 0.99 (0.71 to 1.41) | 0.97 (0.69 to 1.36) | 0.852 |
| 39-48 (39) | 1.69 (1.32 to 2.18) | 1.54 (1.20 to 1.97) | 0.001 |
| >48 (91) | 1.79 (1.46 to 2.20) | 1.56 (1.23 to 1.98) | < 0.001 |
| **Patient with history of toxic substances** |  |  |  |
| None (109) | *Ref* |  |  |
| Smoking (10) | 1.21 | 1.00 to 1.47 | 0.048 |
| **Pre-injury ASA - PS (America Society of Anesthesia - Performance Status)** |  |  |  |
| Normal healthy patient (282) | *Ref* |  |  |
| Patient with mild systemic disease (19) | 1.08 | 0.850 to 1.38 | 0.514 |
| Patient with severe systemic disease (23) | 1.36 | 1.14 to 1.64 | 0.001 |
| **Presence of Swirl sign, n (%)** |  |  |  |
| No (32) | *Ref* |  |  |
| Yes (292) | 2.26 | 1.29 to 3.95 | 0.004 |

| **ICU admission**  Total duration (days) |
| --- |
|  |

                  Type: Numeric (byte)
                 Range: [1,26]                        Units: 1
         Unique values: 15                        Missing .: 291/324
                  Mean:      11
             Std. dev.: 8.65303
           Percentiles:     10%       25%       50%       75%       90%
                              1         4        10        16        25
